# Reimagining the Referral to Wellness Services for Youth with Chronic Conditions as a Conversation: A Human-Centered Approach

**DOI:** 10.3390/healthcare14131866

**Published:** 2026-06-26

**Authors:** Emily von Scheven, Addison Cuneo, Sneha Daya, Bhupinder Nahal, Lydia Tinajero-Deck, Jan Yeager

**Affiliations:** 1 Department of Pediatrics, University of California, San Francisco, CA 94158, USA; addison.cuneo@ucsf.edu (A.C.); bhunahal@gmail.com (B.N.); lydia.tinajerodeck@ucsf.edu (L.T.-D.); 2 Department of Medicine, Georgetown University School of Medicine, Washington, DC 20057, USA; sneha.s.daya@medstar.net; 3 Department of General Internal Medicine, University of California, San Francisco, CA 94158, USA; jan.yeager@ucsf.edu

**Keywords:** wellness, chronic illness, children, Human-Centered Design, conversations

## Abstract

**Highlights:**

**What are the main findings?**

- The optimal referral to wellness services for children with chronic illnesses is more than an administrative act and includes important experiential characteristics.
- Human-Centered Design allowed for the purposeful design of a human conversation to support referrals to wellness services for children with chronic illnesses.

**What are the implications of the main findings?**

- Referrals to wellness services for children with chronic illnesses should take the form of a conversation that is empowering, supportive and hope-inspiring.
- Enhanced attention to wellness for the rising population of children growing up with chronic illnesses has the potential to improve their lives both today, and across their lifetime.

**Abstract:**

**Background/Objectives:** Despite the growing popularity of the topic of wellness in society, children with chronic illnesses are rarely introduced to the concept. Wellness may be an unexpected and complex topic for a medical visit, especially for those living with chronic medical conditions. Our goal is to intentionally design an individualized referral process to wellness services for children with chronic illnesses. **Methods:** Human-Centered Design (HCD) methods were utilized to understand patient, caregiver and provider needs and challenges when making wellness service referrals. Stakeholders participated in workshops and interviews, which informed the design of a referral prototype. The referral prototype was evaluated through simulations and was pilot-tested in a new Center. **Results:** Optimal referrals to wellness services are best delivered through conversations that are compassionate, relational, respectful and motivating. We developed operational, contextual and experiential Design Requirements that informed a personalized “Wellness Conversation” to create an experience that was distinct from a medical visit. The conversation follows a four-step framework: trust and rapport building, assessment of current state of wellness, prioritization of wellness areas, and establishment of goals in wellness planning. **Conclusions:** HCD allowed us to produce a referral prototype with high perceived acceptability, feasibility and fidelity. These findings indicate that approaching referrals to wellness services as a conversation may help create a more positive, supportive, and hope-inspiring experience for children and families.

## 1. Introduction

As advances in healthcare have improved survival for children with previously fatal conditions, the rates of childhood chronic disease have also increased [1]. An estimated 40% of school-aged children and adolescents are affected yet pediatric healthcare remains focused on acute problems rather than proactive, wellness-oriented care [2]. There is an urgent need for innovative models of care that address the unique needs of children facing a lifetime of chronic illness. Enhanced attention to wellness for these young people, many of whom carry their condition with them into adulthood, has the potential to improve lives.

While ad hoc wellness services have emerged at tertiary medical centers caring for youth with chronic medical conditions, comprehensive wellness programs are uncommon. Thus, we recently developed a new Center to holistically address wellness across eight wellness domains: physical, occupational, intellectual, social, emotional, environmental, spiritual and financial. Children with chronic illnesses frequently experience isolation, a depressed mood, challenges with navigating the healthcare system and difficulties with achieving life goals [3,4]. Thus, our Center connects patients with wellness services such as social and emotional support, fitness consultation, nutrition consultation and support for transition from pediatric to adult care. In addition, the Center provides classes and programs that support patients and families coming together in community for shared learning and fun. As we embarked on designing the Center, we recognized that one challenge of integrating wellness programs into the pediatric healthcare system is the referral process, including the manner by which wellness services are introduced to patients. We had observed from prior clinical experience that the usual healthcare referral process, which is reactive to existing problems, and typically involves a brief administrative action by providers, often starts from the position of “something is wrong with you” and thus does not support the positive spirit of wellness. It was critical to the success of the Center and to our goal of helping patients that we design a successful referral process.

An optimal referral is more than the administrative act of placing an order. Recognizing that patients often do not follow through on referrals made in the medical setting [5,6], we sought to design a referral process that considered experiential characteristics, such as how the referral feels, is perceived and is emotionally experienced by patients, in addition to operational aspects. Referrals ideally include an assessment of the patient [7], an understanding of the patient’s goals, a provider’s recommendation and ultimately the act of placing the referral order. We anticipated that the contextual factors underlying this multi-step process were critical to whether or not a wellness service referral was completed, satisfying and effective. Here we present the results of our effort to purposely design a referral process to wellness care, considering all aspects of the process, to ensure success.

Given the absence of standardized guidelines or prototypes for wellness programs in the context of childhood chronic illness care, we chose to utilize a Human-Centered Design (HCD) approach to design our Center. HCD emphasizes the end user’s needs when building a new process. Although HCD has been applied to pediatric health interventions, healthcare transition programs [8,9], mental health services [10] and digital health tools [11], we are not aware of published reports describing the use of HCD and co-design approaches for the development of a referral conversation, referral workflow, and enrollment pathway into a wellness program for youth with chronic conditions. Thus, we chose to utilize the HCD approach to gain an understanding of the optimal patient experience of being connected to wellness services within our tertiary-care academic children’s hospital. We considered a range of stakeholders, including patients, caregivers and healthcare providers. The HCD process is often visually depicted as a double diamond representing alternating phases of divergent (exploring an issue widely) and convergent (taking focused action) thinking and includes four phases: Discover, Define, Develop, and Deliver [12]. The Develop phase includes the creation of a prototype that serves as an early model of a product and allows designers to test ideas, gather user feedback and refine functionalities before finalizing the design. We anticipated that following this process would enable us to develop a service prototype that met the real-life needs of patients, caregivers and providers and ultimately would allow us to successfully connect youth with chronic conditions to our new wellness services. The project originated as a pragmatic service design and program-development effort embedded within the creation of a new Wellness Center rather than as a fully protocolized qualitative research study. As the work progressed, systematic documentation of stakeholder experiences, design decisions, and implementation observations enabled retrospective analysis of the intervention development process and resulting referral framework. Here we report on the innovative approaches that were employed to elucidate the human factors underlying an acceptable referral at our Center, and to design a referral process for connecting children with treatable but often incurable chronic conditions to wellness services.

## 2. Materials and Methods

**Figure 1:**
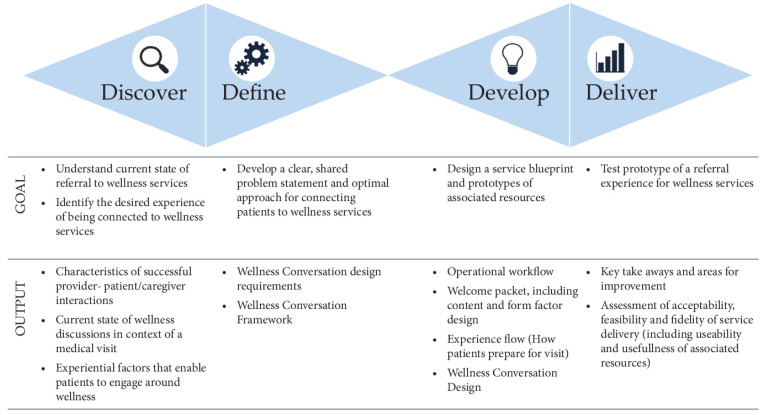
Goals and outputs of the four-step Human-Centered Design process. This figure illustrates the Human-Centered Design process including the goals and outputs of each phase. (1) Discovery; Understand stakeholder experiences and uncover needs in seeking referrals to wellness services. (2) Define; Insights were translated into problem statements, Design Requirements to guide solution development and a framework specifying the components of a Wellness Conversation. (3) Develop; Solutions, including a service blueprint and supporting materials, were generated and iteratively refined. (4) Deliver; pilot-testing of the referral experience to explore acceptability, feasibility and usability. Activities described during the Discover, Define and Develop phases were conducted as part of service design activities, outside of the research environment. The activities described in the Deliver phase were conducted in a clinical environment.

This study was conducted as a human-centered service design and intervention development project that incorporates qualitative and participatory inquiry methods to understand stakeholder experiences and inform the design of a wellness referral process. The methods utilized in each phase of the HCD process are summarized in Figure 1. The primary aim is to develop a contextually grounded referral model responsive to stakeholder needs, workflows, and implementation realities rather than to generate standalone qualitative theory or formally evaluate intervention effectiveness. As is common in HCD practice, inquiry activities evolved iteratively throughout the development process in response to emerging stakeholder insights and operational needs.

### 2.1. Participants and Setting

**Figure 2:**
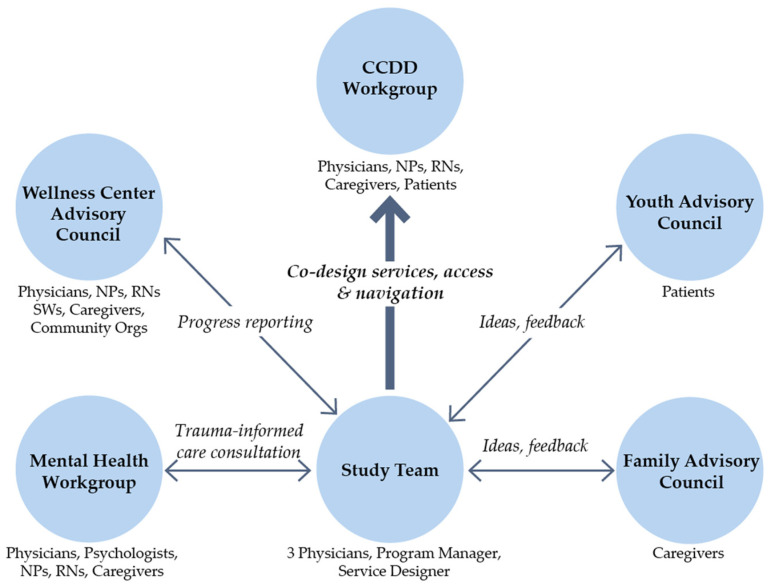
Stakeholder ecosystem. The stakeholder ecosystem allowed the Center to identify insights from diverse perspectives regarding experiences, to brainstorm design features, and to provide feedback on work in progress. The Family Advisory Council and Youth Advisory Council were existing groups in the hospital. Other groups were formed to design the new Wellness Center for Youth with Chronic Conditions. CCDD (Clinical Care Design and Delivery); NP (nurse practitioner); RN (registered nurse); SW (social worker); CLS (child life specialist).

Recognizing that stakeholder engagement is fundamental to the HCD process, we established a stakeholder ecosystem (Figure 2) to solicit insight into experiences, brainstorm design features, and provide feedback on work in progress. The purposeful inclusion of diverse perspectives enabled us to design a solution that addressed the needs of our stakeholders and was practical for real-world implementation. Consistent with HCD methodology, stakeholder engagement prioritized depth of contextual insight and iterative refinement over exhaustive sampling.

Participants included 96 patients, caregivers and providers (physicians, nurses, social workers, child life specialists and transition coordinators). We invited participants from existing groups, including the University of California, San Francisco affiliated Benioff Children’s Hospital (BCH) Family Advisory Council (FAC), BCH Youth Advisory Council (YAC), Wellness Center Advisory Council, Wellness Center Clinical Care Delivery and Design Workgroup, Wellness Center Mental Health Workgroup and Wellness Center Clinic. Not all participants participated in all phases of the project.

### 2.2. Discover Phase

We began with the supposition that as for other referrals in the healthcare setting, a referral to wellness services should start with an assessment of the patient [5]. We suspected that the assessment for a wellness service referral would feel more empowering to patients if guided by the patient’s own preferences and needs. In parallel, early in the Discover phase, we determined that the referral process should take the form of a conversation. Thus, subsequent discovery focused on acquiring an in-depth understanding of the factors involved in a conversational exchange between a provider and patient or caregiver, and how a conversation about wellness might manifest as a referral mechanism. Inquiry addressed the flow of a conversation, characteristics of successful provider–patient interactions, expectation-setting, assessing the current state of wellness in the context of medical visits, and experiential factors that enable patients to engage with wellness. Topics were explored through several rounds of inquiry with diverse stakeholders as follows:

Multi-stakeholder Ideation Workshops. Two workshops were conducted with 10 pediatric healthcare providers (physicians, nurses and social workers), 3 adult medicine physicians, 3 young patients with chronic illness, 2 caregivers of youth with chronic conditions, 1 transition coordinator and 1 child life specialist. To explore “what could be” we brainstormed how we might design a referral that would connect patients to the appropriate wellness services in a way that was flexible, empowering, and longitudinal. The discussion was structured around three questions: (1) What is the right framework for guiding patients to the right wellness services? (2) What underlying information is needed in each part of the framework? And (3) What might the user experience be like?

Multi-stakeholder insight session stage 1. Ten participants (5 physicians, 1 social worker, 1 nurse practitioner, 1 caregiver and 2 service designers) reviewed the results of earlier workshops. We solicited responses to the prompt, “How might providers introduce wellness to a patient?” as part of a conversational model for the referral process.

Provider survey. A web-based survey (Qualtrics) was conducted with physicians, social workers and psychologists (number = 38) to identify current approaches and barriers to conducting Wellness Conversations during a medical visit. Respondents provided free text responses to 3 questions: (1) What words do you use when you introduce a wellness concept to your patients? (2) When you start a conversation with patients surrounding wellness and preventive care, what have you found works well? And (3) When you start a conversation, what are some of the challenges you have had? Additionally, respondents ranked a list of 7 potential challenges in order of importance and voted on potentially helpful patient-facing resources.

Multi-stakeholder insight session stage 2. Ten participants (4 physicians, 1 social worker, 2 nurses, 1 transition coordinator, 1 caregiver and 1 service designer) participated in a second insight session to review the provider survey results and to respond to the following prompts: (1) “How do we frame the conversation to remove the challenges mentioned in the survey?” and (2) “What form and content might a Wellness Conversation prototype take?”

Provider one-on-one interviews. One-on-one interviews were conducted with 17 providers who had completed the provider survey and expressed interest in contributing additional information through an interview. One-hour interviews were conducted on Zoom (JY, AC, SD) to probe further into (1) the circumstances that prompt providers to discuss wellness with youth with chronic conditions; (2) provider approaches to adapting the conversation to suit the spectrum of patient needs and (3) tactics for overcoming common barriers like patient hesitancy.

Patient Focus group. Five youth with chronic illnesses on the BCH YAC participated in a focus group, to explore what the patient/provider interaction should feel like and what conditions are required to enable comfortable and safe sharing of personal information.

Caregiver insight session. Six caregivers on the hospital-wide BCH FAC participated in a 1 h virtual (Zoom) insight session to gather their perspectives on discussing wellness in the context of a medical visit. Three prompts guided the discussion: (1) “If one of your child’s providers addressed wellness in the context of a medical visit, what would you think about it?”; (2) “Who would you like to talk to about this?”; and (3) “How do you know when you have a good fit between you and the provider?”

Multi-stakeholder insight session #3. A 3rd virtual (Zoom) insight session was held to elucidate how to create the optimal conditions for conducting a Wellness Conversation. A total of 17 individuals (5 physicians, 3 nurses, 1 social worker, 1 psychologist, 1 transition coordinator, 1 patient advocate, 2 service designers and 3 caregivers) were divided into 3 groups to discuss 7 contextual factors relevant to conducting a Wellness Conversation in the context of a medical encounter: provider/patient fit, time, perception of role and whose job it is, provider factors, patient/family factors, external factors and institutional factors. These contextual factors emerged from earlier inquiry findings.

### 2.3. Define Phase

Data from the Discover phase were analyzed to identify patterns, needs, and key stakeholder considerations. These insights were used to define the referral process and inform Design Requirements, prototype development, and a structured Wellness Conversation Framework to support referral conversations.

### 2.4. Develop Phase

#### 2.4.1. Prototype Development

A multi-component service blueprint for a wellness referral experience was developed through rapid prototyping to define operational workflows and patient experience flow. Input was collected from all stakeholders, and prototyping considered the diverse follow-up needs of the patient population. A patient/family Welcome Packet was developed. Design features were informed by the Design Requirements and Wellness Conversation Framework.

#### 2.4.2. Prototype Testing

Prototype testing of the Wellness Conversation was conducted through simulation to assess usability, feasibility and the overall experience for patients, caregivers and providers. Four pediatric patients with chronic medical conditions and their caregivers that had participated in earlier phases of work, or who were being cared for by one of the providers on the project team, participated. Patients included an 8-year-old White male with juvenile dermatomyositis, a 12-year-old White female with epilepsy, a 15-year-old Black female with chronic headaches and a gastrointestinal disorder and a 21-year-old Asian male with developmental delay. Simulations were conducted in English. Three providers (a clinical social worker, an integrative medicine pediatrician, and a dual-boarded medicine-pediatric physician) from the Wellness Center Advisory Council acted as the wellness provider during the simulations. An orientation session one week before the first scheduled simulation helped to familiarize providers with objectives, Wellness Conversation resources and patient experience flow.

Patients and caregivers were sent the Welcome Packet 5 days in advance of the visit via mail. The packet included directions on how to complete the pre-visit activities including reading a Wellness Booklet to familiarize themselves with the topic and completing the maps and worksheet. They were instructed to take photographs of the completed materials and submit the images to the provider. The providers were instructed to review the submitted materials before the visit. During the 1 h virtual (Zoom) simulation, providers attempted to use the maps and worksheet as conversation prompts while following the Wellness Conversation Framework. The sessions were recorded and subsequently reviewed by the project team.

A debrief was conducted by the project team with patients and caregivers 2–3 days after the simulation to collect feedback on their experience and to understand the usability of the resources. Data collected during feedback included their experience of receiving and interacting with the Welcome Packet, understandability of instructions, feasibility and experience of the process (e.g., ability to fit pre-work into the context of home life; ability to successfully photograph and send materials to the provider prior to the visit), and the conversation experience (e.g., did it meet patients’ expectations, were providers able to follow the framework, effectively use the materials as prompts, and reach a wellness goal within the 1 h timeframe).

The provider debrief focused on the timing of receiving completed maps and worksheet; how well-prepared they felt; ease of using the materials to prompt and guide the conversation; ease of following the Wellness Conversation Framework; responsiveness of patients to the conversation; and challenges engaging with patients/caregivers.

### 2.5. Deliver Phase

The entire service as described in the service blueprint, including the Wellness Conversation, was implemented in the clinical environment at the BCH Wellness Clinic during 90 min new patient visits. Implementation-phase observations and stakeholder feedback were used formatively to explore perceived acceptability, feasibility, and fidelity of the Wellness Conversation delivered during pilot-testing. These assessments were based on stakeholder feedback, 1-on-1 provider interviews, and consistency with Design Requirements as discussed at staff meetings, rather than formal quantitative implementation instruments.

### 2.6. Research Team and Reflexivity

The study team included pediatric subspecialists, service design contributors and health systems personnel involved in development of the Wellness Center. Recognizing that these individuals valued wellness-oriented care and could influence data collection and interpretation, findings were reviewed across stakeholders and iteratively revisited during stakeholder engagement activities to assess resonance with participant experiences, challenge assumptions and contextualize emerging interpretations.

### 2.7. Data Analysis

Qualitative materials generated through interviews, workshops, insight sessions, focus groups and simulations were synthesized using an iterative interpretive approach consistent with HCD practice. Facilitator notes, workshop outputs and survey responses were reviewed in detail by members of the project team. One project team member (JY) with expertise in HCD research and synthesis led the iterative thematic synthesis. Recurrent experiential concerns, workflow barriers, communication patterns, contextual tensions, and stakeholder needs relevant to the wellness referral experience were identified and iteratively organized into emergent insight areas and design opportunity domains. Preliminary interpretations and proposed design implications were discussed with the broader study team and revisited during subsequent stakeholder engagement activities, allowing refinement and confirmation of resonance with participant experiences.

Provider survey data was analyzed using summary statistics. An exploratory implementation evaluation considering acceptability, feasibility and fidelity [13] was conducted during the Deliver phase using systematic synthesis of stakeholder feedback.

Analysis focused on generating operational, contextual, and experiential Design Requirements to inform intervention development rather than development of formal explanatory qualitative theory.

Credibility was strengthened through triangulation across stakeholder groups, inquiry formats, and iterative phases of engagement.

## 3. Results

### 3.1. Stakeholder Findings (Discover Phase)

Insights from the ideation workshops revealed the importance of structuring the referral process as a conversation rather than a check-the-box action. Participants emphasized that a conversational approach would make the process feel more human, relational, and respectful. Without these qualities, wellness services risk being perceived as unnecessary or unwelcome, reducing the likelihood of their success. We thus set about to design a conversation, something that we all do intuitively, every day, but if designed purposefully could support a successful referral process and outcome for patients. We named this process a Wellness Conversation and we focused our subsequent stakeholder engagement activities on understanding the elements of a successful conversation so that we could ensure consistency and develop training and supportive tools.

Designing a referral as a conversation, rather than the typical medical intake Q&A that takes place in one compressed moment in time and is driven and controlled by the provider, required focusing on both the tools and the interaction around them. Research on relationship-centered communication has demonstrated that asking open-ended probing questions, using layered follow-up questions, and eliciting patient concerns in stages yields better communication outcomes [14,15]. The social penetration theory or “peeling the onion” model [16] for a conversation is a metaphor that describes how relational depth and meaning develop gradually through successive layers of disclosure, understanding, and trust, rather than all at once. Based on what we heard from the stakeholders, we decided to incorporate these approaches into the design of the Wellness Conversation.

Provider surveys revealed considerable variation in how they were already addressing wellness during their visits with young people. Approaches included asking opening questions, normalizing wellness challenges, and offering a general description of wellness. Despite the varied approaches, there were common themes, including focusing on long-term goals, sources of joy and self-care; and drawing on motivational interviewing and creating a positive patient experience in order to build rapport. In rank analysis from the provider survey, the top three significant provider barriers to engaging youth with chronic conditions in a conversation about wellness were: (1) competing priorities involving the patient’s medical condition; (2) mental health and social contextual factors and (3) limited provider time. Regarding potentially useful patient-facing resources for overcoming these barriers, videos introducing wellness were the most frequently selected resource (25/38, 66%), followed by interactive online modules (23/38, 61%) and booklets/brochures (19/38, 50%); multiple selections were permitted.

The one-on-one provider interviews clarified the flow of a successful provider–patient conversation about wellness by identifying three key phases: (1) introducing the notion (e.g., what conditions trigger a conversation about wellness; how is wellness situated); (2) moving the conversation along (e.g., engaging patients to get buy-in and set wellness goals); and (3) making the referral to wellness services. Central to introducing the notion is finding an opening in the context of the visit. Moving the conversation along requires successfully building trust with the patient/family and empowering them. Finally, a successful referral also requires a good handoff between the person conducting the Wellness Conversation and the wellness service provider.

Patient and caregiver participants emphasized the importance of trust, rapport building, comprehension and the feeling of connection. Providers identified the importance of assessing a patient’s current state of wellness across all domains of wellness. Also important was the patient’s ability to set goals and situate the conversation and the goals in the context of their own life and state of health. Caregivers identified the importance of having a skilled provider conduct the conversation, who is also a “good fit” for the family. Caregivers also emphasized that questions about mental health are sensitive and necessitate a demonstration of empathy by the provider and acknowledgement that this may be a very challenging time for them. Several participants expressed concerns about time and timing, as in-depth conversation can take a long time compared to some medical visits.

### 3.2. Derived Design Requirements and Framework (Define Phase)

Insights from discovery were used to generate Design Requirements and a four-part Wellness Conversation Framework that ensures both a meaningful connection between the provider and the patient and/or caregiver, and a Wellness Conversation that converges on identifying patient-selected wellness goals and actions.

#### 3.2.1. Design Requirements

**Figure 3:**
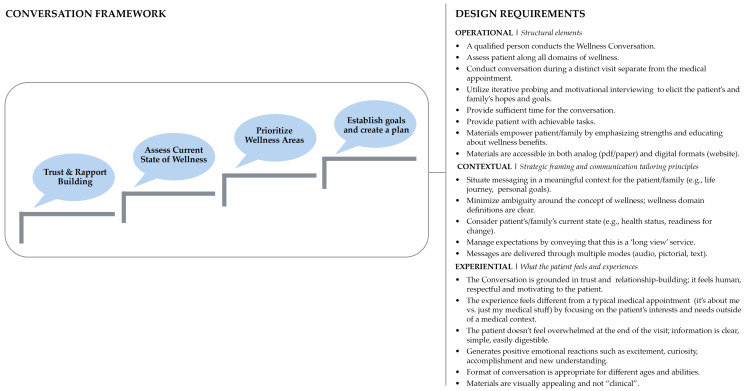
Conversation Framework and Design Requirements. A 4-step framework provides structure for the provider, ensuring it stays focused on what is meaningful to the patient while acquiring the information needed for developing a wellness plan and an appropriate referral. The conversation and referral process are based on a set of Design Requirements addressing operational, contextual and experiential aspects of the service.

Analysis of insights gained from the Discover phase were used to define operational, contextual and experiential Design Requirements (see Figure 3). Operational Design Requirements address process, personnel and materials needed to conduct a successful Wellness Conversation. Contextual Design Requirements address strategic framing to ensure that the conversation is situated in the patient’s context and makes clear the expectation that this is a “long view” service focused on building a lifetime of wellness. Experiential Design Requirements address esthetics of materials and how the Wellness Conversation should feel for a patient and family, with emphasis on feeling different from a typical medical appointment.

All stakeholders agreed that successful discussions about wellness between patients and providers require trust, relationship-building, and iterative probing in order to learn the patient’s and family’s wishes and goals, and to position the referral around hope and possibility. Success depends on creating an experience that feels different from a typical medical visit by focusing on the patient’s interests and needs outside of the medical context. Patients suggested that this could be achieved by ensuring the process was interesting, and more about “me” rather than “just my medical stuff”. This desire extended to materials which patients and caregivers wished to be visually appealing and not “clinical” in appearance.

For some patients the concept of wellness may be new or confusing, especially for those living with chronic medical conditions. Furthermore, a direct discussion about wellness may be unexpected in the context of a medical visit. Thus, providing a clear explanation about the wellness domains and ensuring that there is no ambiguity about why wellness is being discussed in the medical visit is important. Participants suggested that the concept of wellness could be introduced with questions such as “What makes you happy?”, “How can I support you?”, “What does wellness mean to you?”, or “What are the things that you want to be doing that you aren’t doing?” To address the concern that for some patients the concept of wellness may not seem “relevant” or important in the context of illness, participants suggested that the concept be framed as, despite illness. There may be an opportunity for some wellness and prevention, in addition to what they are already experiencing. Another approach is to address the patient’s sense of belonging as a path to wellness. This could be achieved through questions such as “So how are you?” or “I want to make sure this visit is more productive for you than for me.”

We learned that the Wellness Conversation should take place at a planned time during a distinct visit separate from the medical appointment. This overcomes the barrier of limited provider time and conflicting priorities. We suspect that conducting the Wellness Conversation separate from a usual medical encounter would allow sufficient time for providers to deliver information in a way that accommodates different ages, abilities, and learning styles; connect with patients to ensure a positive emotional experience; and co-create a personalized wellness plan with goals that feel achievable. Having a distinct context for the Wellness Conversation also better situates the content for caregivers and patients. The conversation should be conducted by a qualified person, ideally with skills and knowledge about shared decision-making (SDM), stages of change psychology, motivational interviewing, and trauma-informed care.

#### 3.2.2. Wellness Conversation Framework

Insights about the movement or flow of a typical conversation were used to construct a four-step Wellness Conversation Framework to provide structure for the provider and guarantee consistency (Figure 3). Step 1, Trust and rapport building includes building connection and ensuring patient understanding and comfort. Step 2, Assessing current state of wellness, the patient describes their needs and envisions an improved future state. Step 3, Prioritization of wellness areas, the patient shares their own priorities. Step 4, Establishing goals and creating a plan, the patient sets their own goals and expresses empowerment and feeling supported. The Wellness Conversation should embody the Design Requirements. The experience should feel different from a usual medical visit and elicit positive emotional reactions.

The Wellness Conversation should follow the usual phases of a human conversation: introduction of a notion, moving the conversation along and making the referral. The Wellness Conversation Framework ensures that the conversation feels “natural” while also making sure that critical elements are addressed every time, for every patient. Finding an opening when introducing the notion of wellness may be initiated by a patient who asks about wellness or topics other than their medical condition, or it may be initiated by a caregiver who asks about integrative medicine, or social services for example. But if not initiated by the family, then the provider is obligated to find the opening and introduce the topic. The second phase, moving the conversation along, which is characterized by building trust and empowering families, can be achieved by establishing common ground (e.g., seeing the patient beyond their medical condition, directing questions to patients and not only caregivers), ensuring continuity (e.g., having a consistent point person so the patient does not need to repeat their story), demystifying ideas and reducing complexity (e.g., using simple words or more relevant words, e.g., ask how can we make your life better rather than using the word wellness), setting expectations (clarifying what will happen at this visit versus the next, and timing) and being flexible (meeting the patient where they are, considering the state of health and family life).

Establishing empowerment for patients and caregivers is facilitated by helping patients to identify their support system, giving caregivers permission to focus on their own wellbeing, and helping kids to take ownership of their own bodies and health. Central to the act of making the referral for wellness services is ensuring a good handoff. This can be achieved by ensuring that the handoff is to a trusted professional and addressing feelings of abandonment.

### 3.3. Prototype Development and Simulation Findings (Develop Phase)

**Figure 4:**
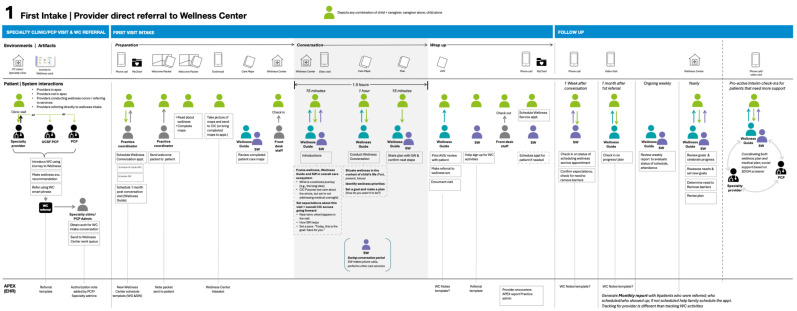
Service blueprint. The service blueprint visually describes the architecture of the initial intake with the flow of activities, interactions between patients and different points of contact, and supporting components. The gray area depicts the Wellness Conversation.

**Figure 5:**
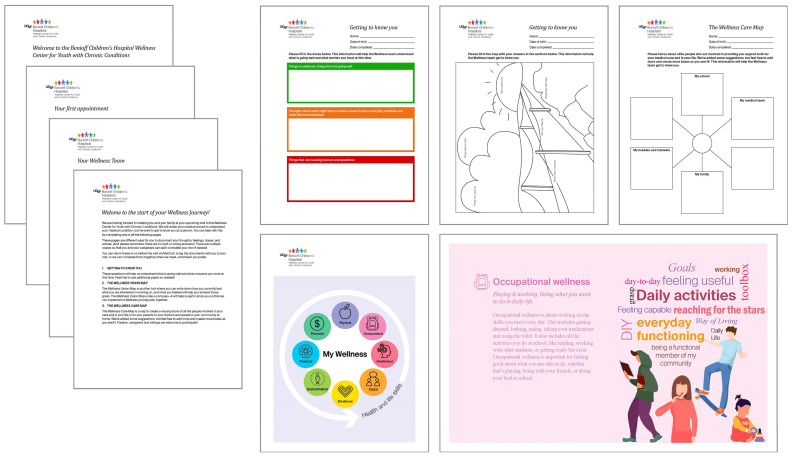
Welcome Packet. Components include orientation material, pre-visit maps and worksheet, and a booklet explaining the eight domains of wellness.

A service blueprint (Figure 4) depicting the operational workflows and a Welcome Packet (Figure 5) with resources were developed to support a referral to wellness services.

#### 3.3.1. Welcome Packet

The prototype for a patient/family Welcome Packet includes the following components: Welcome letter, Frequently Asked Questions, wellness team bios and headshots, instructions about the packet, a Wellness Booklet with plain-language information about wellness including definitions of eight wellness domains that are used by the Wellness Center, Vision Map, Care Map and a Getting to Know You worksheet (Figure 5). The packet design is grounded in the constructs that will be discussed in the Wellness Conversation and supports the patient and family in engaging with the provider around what wellness means to them in the context of their lives and their illness experience.

The first pre-visit activity is a vision-mapping exercise, which uses a visual picture of a bridge leading to a sunset with text prompts: “how I want to be”, “things I’m good at”, “things I don’t like”, “things that are hard”, “what I wish I could do”, and “who can help me.” The design reflects the themes shared by providers when asked how they start a Wellness Conversation: inquiry into long-term goals, sources of joy, and self-care. In the second activity, the care map (modified from Cristin Lind [17,18]), the patient draws a map of all the people who support them in their life, such as teachers, community groups, family members and friends. An example is given, followed by a blank page for the patient to create their own map. The purpose of the care map is to (1) decrease the burden of information exchange between provider and patient, (2) allow easy identification of existing resources that could be leveraged towards wellness goals, and (3) situate the wellness team in the care ecosystem during the Wellness Conversation.

The operational workflow describes the following flow of activities: patients are provided a wellness Welcome Packet by mail or by downloading from the Wellness Center website, patients complete the care map, vision map and Getting to Know You worksheet, patients upload a photograph of the materials through the patient portal, materials are reviewed by the provider before the visit, and the provider refers to completed materials during the Wellness Conversation to facilitate the discussion.

#### 3.3.2. Prototype Testing Results

Of the four patients who participated in a simulated Wellness Conversation, three completed the pre-work and the Wellness Conversation with a provider. Patients and caregivers reported that the pre-work materials in the Welcome Packet were easy to access, the content was easily understood and educational, and the materials were visually pleasing. However, they had difficulty finding the time to complete the pre-work in advance of the conversation and in some cases expressed concern about not doing the activities “correctly”. They also reported that the concept of wellness was novel and difficult to connect with amidst the medical context of their underlying health condition. Providers reported that they would have liked to have more time to review the maps and Getting to Know You worksheet in advance of the conversation. And they would have benefited from having more of the patient’s medical background prior to the encounter in order to better identify resources for the patient.

### 3.4. Exploratory Implementation Observations (Deliver Phase)

The Wellness Conversation was further evaluated with 156 patient/caregiver dyads over 6 months in clinical practice. Patient demographics were as follows: average age 14.9 years (range 3–23 years), mixed gender (56% female) and mixed race/ethnicity (35% Caucasian, 34% LatinX, 10% Asian, 3% African American, 18% mixed/other/unknown). There was high perceived acceptability across all stakeholder groups with patients and caregivers expressing high levels of appreciation for this service. All patients successfully developed wellness goals. Caregivers stated, “I’ve never had an hour where I’m asked about how I am doing”, “My [child] left that meeting energized and feeling hopeful—which has not been the case in a long time”, and “I feel a glimmer of hope for the first time in a long time”. Feasibility was good, with the majority of challenges relating to scheduling and the expectation that patients would complete the maps and worksheet in advance of the visit. Despite this, implementation was generally feasible within this setting and providers adapted their workflow so that the pre-work was not needed for the majority of patients. Despite several challenges, providers reported that “We really talked about all different kinds of things, all different areas of a person’s life. We talked about all the eight wellness domains.” Fidelity to core components, including the Design Requirements and Wellness Conversation Framework, was maintained. Patients consistently described that the visit was what they have been needing all along. Many reported that this was the first time they felt heard and stated, “this is the first time anybody had asked them that question.”

## 4. Discussion

This was a real-world service design effort conducted in a complex clinical environment that aimed to elucidate the optimal process for connecting young people living with chronic medical conditions and their families to wellness services. The wellness services at our new Center provide an extra layer of help and support beyond standard medical care, to allow young people living with chronic medical conditions to thrive despite their underlying illness. A central contribution of this work is the reconceptualization of referral as a relational and experiential process rather than solely an administrative act. The Wellness Conversation Framework suggests that referral success may depend not only on operational workflow, but also on the emotional, interpersonal, and contextual conditions under which wellness services are introduced to a patient. We learned that successful discussions about wellness between patients and providers require trust, relationship-building, and the use of iterative probing to learn the patient’s and family’s hopes and goals. This led to the insight that the referral process could be reimagined as a conversation. A conversation allows for a smooth natural progression of exchange of ideas between the provider and patient or caregiver so that everyone feels engaged and the provider is able to successfully guide the patient into a receptive state of mind, where they become open to engaging with the topic of wellness despite their experience of life with a medical condition. The deconstruction of an activity performed intuitively by all of us all the time, a conversation, into its core elements, allowed us to prospectively design a referral process that had the elements of a successful human conversation. The four core elements are represented in the Wellness Conversation Framework that we developed. Although there has been extensive research on conversation analysis and patient–provider communication around referrals [19], to our knowledge there have been no prior reports re-conceptualizing wellness service referrals as a conversation. Our results are consistent with prior reports demonstrating that provider–patient conversations can function as dialogic exchanges of information and meaning, not just transmission of facts or instructions [20]. Building trust and rapport, which can be achieved through conversation, is essential for SDM and has been shown to impact numerous outcomes including health status [21]. The literature related to patient–provider communication has largely focused on communications underlying medical testing and treatment decision-making, but not specific to referrals. In this report we provide preliminary evidence of feasibility, acceptability and stakeholder support for an intentionally designed Wellness Conversation developed to connect patients to wellness services at our Center. We anticipate that the Wellness Conversation Framework could also be applied to other critical conversations in the healthcare setting.

Several novel Design Requirements for the desired experience emerged through this work. The desire for the experience to feel different from a typical medical visit was mentioned by multiple participants. Patients suggested that this could be achieved by making sure the process was interesting, and more about “me” rather than “just my medical stuff”. This desire extended to materials which patients and caregivers hoped would be visually appealing and not “clinical” in appearance. Another salient Design Requirement was that the concept of wellness be clear, especially for an individual living with a chronic medical condition. The idea of wellness may be new to young people, and a direct discussion about wellness may not be expected in the medical context. Thus, explaining wellness topics and the overall visit purpose as part of expectation-setting would be critical to any wellness program procedures or materials. Finally, the need to focus on the patient’s interests and needs outside of a medical context was identified as a critical Design Requirement.

We learned that although some providers already have conversations about wellness with their patients, others do not. Our goal was to ensure access to wellness services for all patients at our Center. Thus, we aimed to develop resources and a well-defined process for providers to follow to ensure standardization of the experience for all users and improved access for patients. The Wellness Conversation Framework scaffolds the Wellness Conversation in a way that feels “natural” while also making sure that critical elements are addressed every time, for every patient. This framework can serve as an educational resource and tool for providers going forward, especially those with less experience discussing wellness with their patients. Despite the intuitive nature of a conversation, as for other forms of communication in the healthcare setting [22], we learned that training is important when implementing a novel process. Successful discussions about wellness between patients and providers are more likely with a skilled provider. The practice of SDM [23], which is grounded in empathy and patient centeredness, can be leveraged throughout the Wellness Conversation and we recommend that all providers have competence in SDM. We also recommend that providers are familiar with the Transtheoretical Model describing the stages of Change [24]. This model looks at a patient’s readiness to embrace a new behavior and can be used at various points in the Wellness Conversation to assess patient motivation for change, especially as recommendations for wellness services are provided. Finally, use of motivational interviewing methods [25] can support meeting several of the Design Requirements by supporting a focus on patient autonomy, embracing a spirit of collaboration between providers and patients, and encouraging providers to evoke the patient’s inner motivation for change. Empowering patients and their families through motivational interviewing methods can ensure that the provider and patient discuss the issues that matter most to the patient.

By using a Wellness Conversation format, we have designed a referral experience that shifts away from the negative framing that typically accompanies healthcare referrals placed in reaction to existing problems or adverse circumstances. We hypothesize that this reframing will improve referral completion rates beyond those commonly reported [5,6,7]. Furthermore, because a patient’s psychological readiness and general state of mind at the point of referral are critical to engagement with wellness services [19,20], our findings suggest that exploring the patient’s attitudes toward help and prior experiences through conversation, as in our design, will support promising referral experiences across diverse patient populations.

The Wellness Conversation simulation revealed several challenges with our prototype. There was tension between the provider’s needs and the patient’s needs, in terms of the topics each wanted to discuss. Some patients felt pressured, feeling that they needed to get wellness “right”. We addressed this during the subsequent testing by emphasizing the importance of setting expectations at the beginning that were patient- and family-driven, rather than provider-driven. Additionally, none of the providers who conducted the simulation were able to create a wellness plan with their patient within the one-hour visit. This was addressed by increasing the visit time to 90 min in the Deliver phase which resulted in successful wellness goal development across all patient encounters. Ultimately, our pilot-testing in the clinic demonstrated that with the use of a HCD approach we were able to produce a prototype for a referral that demonstrated acceptability, feasibility and fidelity.

There are several limitations to consider when interpreting our findings. First, this work was conducted at a single academic pediatric center, which limits the generalizability of findings. The referral workflows, organizational culture, and available wellness resources at our site may differ from those in other clinical settings. And thus, adaptation for community-based or resource-constrained settings will require further study. Second, while HCD methods are well-suited for generating contextually grounded solutions, they prioritize depth of insight over breadth of sampling. As a result, the number of participants engaged across patients, caregivers, and providers was intentionally limited and may not capture the full range of perspectives, particularly from individuals less likely to participate in research or those with competing social or clinical burdens. Furthermore, because this work was conducted as an iterative service design and intervention development effort, formal qualitative coding and inter-coder reliability procedures were not employed. Findings should therefore be interpreted as design-oriented synthesized insights intended to support contextual intervention development rather than exhaustive qualitative thematic analysis. Third, the iterative and participatory nature of the design process introduces potential selection bias, as participants who engaged in interviews and co-design activities may have been more motivated or available than the broader population. This may have influenced both the identification of needs and the refinement of proposed solutions. Future work includes prospectively evaluating the feasibility, acceptability and impact of this newly designed Wellness Conversation referral process across diverse healthcare settings, assessing impact on wellness outcomes and implementing iterative changes as needed to optimize the process.

Despite these limitations, our findings add significantly to the literature by bringing attention to the concept of wellness for children with chronic illnesses, many of whom will carry their illness with them into adulthood. The state of mind of the patient when presenting to wellness services is critical to their engagement and success. Here we provide an innovative path for converting referrals, which are typically an administrative task, to a relational activity which can foster the desired state of mind. In addition to the intrinsic therapeutic potential of the Wellness Conversation, the experience can help patients see the possibility of wellness in the context of their underlying medical condition, perhaps for the first time.

## 5. Conclusions

Despite the growing popularity of wellness in society, children with chronic illnesses are rarely introduced to the concept. This is problematic as the rates of childhood chronic illness continue to rise, thus leaving many children without access to wellness services. The goal of our newly formed Wellness Center for Youth with Chronic Conditions is to structure initial conversations in a way that is personalized and supports psychological readiness and hope. A referral is a critical moment in time, and like other moments it can be intentionally designed to ensure that it is optimized for the users involved. Operations are often the primary focus during new program development. In contrast, here we report how we focused on the human experience as an approach for making systems better. Through our exploration of the characteristics of a referral process we learned that, when successful, it most closely resembled a human conversation. Successfully implemented, a conversation can build trust that helps identify wellness needs of patients and families and connect them with services through the lens of positivity. Within our tertiary pediatric care setting, the Wellness Conversation appeared feasible and acceptable as a human-centered referral approach for connecting youth with chronic illnesses to wellness-oriented services. Our findings have been used to inform the design of our new Center’s innovative program and can also be applied to the design of other critical conversations in the healthcare setting. Additional prospective evaluation is needed to assess transferability, implementation outcomes, referral completion, and patient wellness outcomes across broader healthcare environments. Finally, in addition to generating useful information about how to optimize wellness, our work highlights the value of making intuitive subconscious activities, even those that we do every day like having a conversation, purposefully, as a strategy for ensuring their success.

## Data Availability

The data presented in this study are available on request from the corresponding author. The data are not publicly available due to privacy and ethical restrictions.

## Abbreviations

SDM	Shared Decision-Making
HCD	Human-Centered Design
FAC	Family Advisory Council
CCDD	Clinical Care Design and Delivery
NP	Nurse Practitioner
RN	Registered Nurse
SW	Social Work
CLS	Child Life Specialist
